# Evaluation of an automated feedback intervention to improve antibiotic prescribing among primary care physicians (OPEN Stewardship): a multinational controlled interrupted time-series study

**DOI:** 10.1128/spectrum.00017-24

**Published:** 2024-02-27

**Authors:** Jean-Paul R. Soucy, Marcelo Low, Kamal R. Acharya, Moriah Ellen, Anette Hulth, Sonja Löfmark, Gary E. Garber, William Watson, Jacob Moran-Gilad, Nadav Davidovitch, Tamar Amar, Janine McCready, Matthew Orava, John S. Brownstein, Kevin A. Brown, David N. Fisman, Derek R. MacFadden

**Affiliations:** 1 Division of Epidemiology, Dalla Lana School of Public Health, University of Toronto, Toronto, Ontario, Canada; 2 Chief Physician’s Office, Clalit Health Services, Tel Aviv, Israel; 3 Department of Population Medicine, University of Guelph Ontario Veterinary College, Guelph, Ontario, Canada; 4 Department of Health Policy and Management, Guilford Glazer Faculty of Business and Management and Faculty of Health Sciences, Ben-Gurion University of the Negev, Beer Sheva, Israel; 5 The Public Health Agency of Sweden, Stockholm, Sweden; 6 Public Health Ontario, Toronto, Ontario, Canada; 7 Department of Family and Community Medicine, University of Toronto, Toronto, Ontario, Canada; 8 Department of Health Policy and Management, School of Public Health, Faculty of Health Sciences, Ben-Gurion University of the Negev, Beer Sheva, Israel; 9 Department of Epidemiology, Biostatistics, and Community Health Sciences, Ben-Gurion University of the Negev, Beer Sheva, Israel; 10 Division of Infectious Diseases, Department of Medicine, Michael Garron Hospital, Toronto, Ontario, Canada; 11 Barrie and Community Family Health Team, Barrie, Ontario, Canada; 12 Computational Epidemiology Lab, Boston Children’s Hospital, Boston, Massachusetts, USA; 13 Clinical Epidemiology Program, The Ottawa Hospital Research Institute, Ottawa, Ontario, Canada; MultiCare Health System, Tacoma, Washington, USA

**Keywords:** antimicrobial stewardship, audit and feedback, antibiotic prescribing, primary care, interrupted time series

## Abstract

Tools to advance antimicrobial stewardship in the primary health care setting, where most antimicrobials are prescribed, are urgently needed. The aim of this study was to evaluate OPEN Stewarship (Online Platform for Expanding aNtibiotic Stewardship), an automated feedback intervention, among a cohort of primary care physicians. We performed a controlled, interrupted time-series study of 32 intervention and 725 control participants, consisting of primary care physicians from Ontario, Canada and Southern Israel, from October 2020 to December 2021. Intervention participants received three personalized feedback reports targeting several aspects of antibiotic prescribing. Study outcomes (overall prescribing rate, prescribing rate for viral respiratory conditions, prescribing rate for acute sinusitis, and mean duration of therapy) were evaluated using multilevel regression models. We observed a decrease in the mean duration of antibiotic therapy (IRR = 0.94; 95% CI: 0.90, 0.99) in intervention participants during the intervention period. We did not observe a significant decline in overall antibiotic prescribing (OR = 1.01; 95% CI: 0.94, 1.07), prescribing for viral respiratory conditions (OR = 0.87; 95% CI: 0.73, 1.03), or prescribing for acute sinusitis (OR = 0.85; 95% CI: 0.67, 1.07). In this antimicrobial stewardship intervention among primary care physicians, we observed shorter durations of therapy per antibiotic prescription during the intervention period. The COVID-19 pandemic may have hampered recruitment; a dramatic reduction in antibiotic prescribing rates in the months before our intervention may have made physicians less amenable to further reductions in prescribing, limiting the generalizability of the estimates obtained.

**IMPORTANCE:**

Antibiotic overprescribing contributes to antibiotic resistance, a major threat to our ability to treat infections. We developed the OPEN Stewardship (Online Platform for Expanding aNtibiotic Stewardship) platform to provide automated feedback on antibiotic prescribing in primary care, where most antibiotics for human use are prescribed but where the resources to improve antibiotic prescribing are limited. We evaluated the platform among a cohort of primary care physicians from Ontario, Canada and Southern Israel from October 2020 to December 2021. The results showed that physicians who received personalized feedback reports prescribed shorter courses of antibiotics compared to controls, although they did not write fewer antibiotic prescriptions. While the COVID-19 pandemic presented logistical and analytical challenges, our study suggests that our intervention meaningfully improved an important aspect of antibiotic prescribing. The OPEN Stewardship platform stands as an automated, scalable intervention for improving antibiotic prescribing in primary care, where needs are diverse and technical capacity is limited.

## INTRODUCTION

Antimicrobial resistance is a major global health challenge, leading to untreatable infections, rising health-care costs, and hindering efforts to reduce poverty (1, 2). In 2019, it was estimated that 1.27 million deaths were directly attributable to antimicrobial resistance in bacterial infections (3). With current trends, the expected mortality burden of antimicrobial resistance is expected to grow dramatically over the coming decades (4). Given the slow pace of antibiotic development since the 1980s (5), it is critical to preserve the efficacy of existing drugs by reducing inappropriate prescribing.

Antimicrobial stewardship refers to the effort to optimize the selection, dose, and duration of antimicrobial therapy while minimizing adverse effects and the spread of antimicrobial resistance (6). Stewardship interventions take many forms, including measures targeting providers (e.g., audit and feedback, decision support tools) (7–9), laboratories (e.g., selective reporting of susceptibility testing results), and hospital formularies (6). Feedback interventions (such as reports comparing the prescribing rate of providers to their peers) have been shown to be effective across a variety of clinical settings (7, 10–15).

While significant progress has been made in hospital-based stewardship in recent years, further improvement is needed for antimicrobial stewardship in the community setting (16–18), where the vast majority of antimicrobials for human use are prescribed [e.g., over 90% in Canada (19)] and inappropriate prescribing is common (20–22). The U.S. Centers for Disease Control and Prevention identifies individualized tracking and reporting as a key element of outpatient stewardship, but the lack of capacity and information technology support remains a major barrier (23, 24). To address this deficit, we developed OPEN Stewardship (Online Platform for Expanding aNtibiotic Stewardship), a web-based platform capable of generating automated, personalized feedback reports based on local prescribing data (25–27). Following a One Health approach, the platform was developed for use in both human and veterinary care providers. In this study, we describe a quasi-experimental study of the OPEN Stewardship platform on antibiotic prescribing among primary care providers in Canada and Israel.

## MATERIALS AND METHODS

### Study design

This study used a controlled interrupted time-series design to assess the impact of an automated feedback intervention on prescribing rates and duration of therapy while accounting for secular trends (28). The intervention and recruitment process are described in detail in our published protocol (25) and summarized below; changes from the protocol are outlined in the Supplementary Methods.

### Setting and participants

We enrolled primary care physicians in Ontario, Canada and Southern Israel. Canadian participants were recruited from primary care practices sharing data with the University of Toronto Practice-Based Research Network (UTOPIAN), which included over 500 physicians as of Q4 of 2019 (29). Israeli participants were recruited from Clalit Health Services (CHS), the largest of Israel’s four nationally mandated health maintenance organizations, which covers the majority of residents in Southern Israel through a network of family care practices. Physicians were eligible for enrollment in the intervention if they had prescribing data available in the UTOPIAN or CHS databases; non-participating physicians with prescribing data in the UTOPIAN and CHS databases were used as controls. Further details on recruitment are available in the Supplementary Methods.

### Intervention

**Fig 1:**
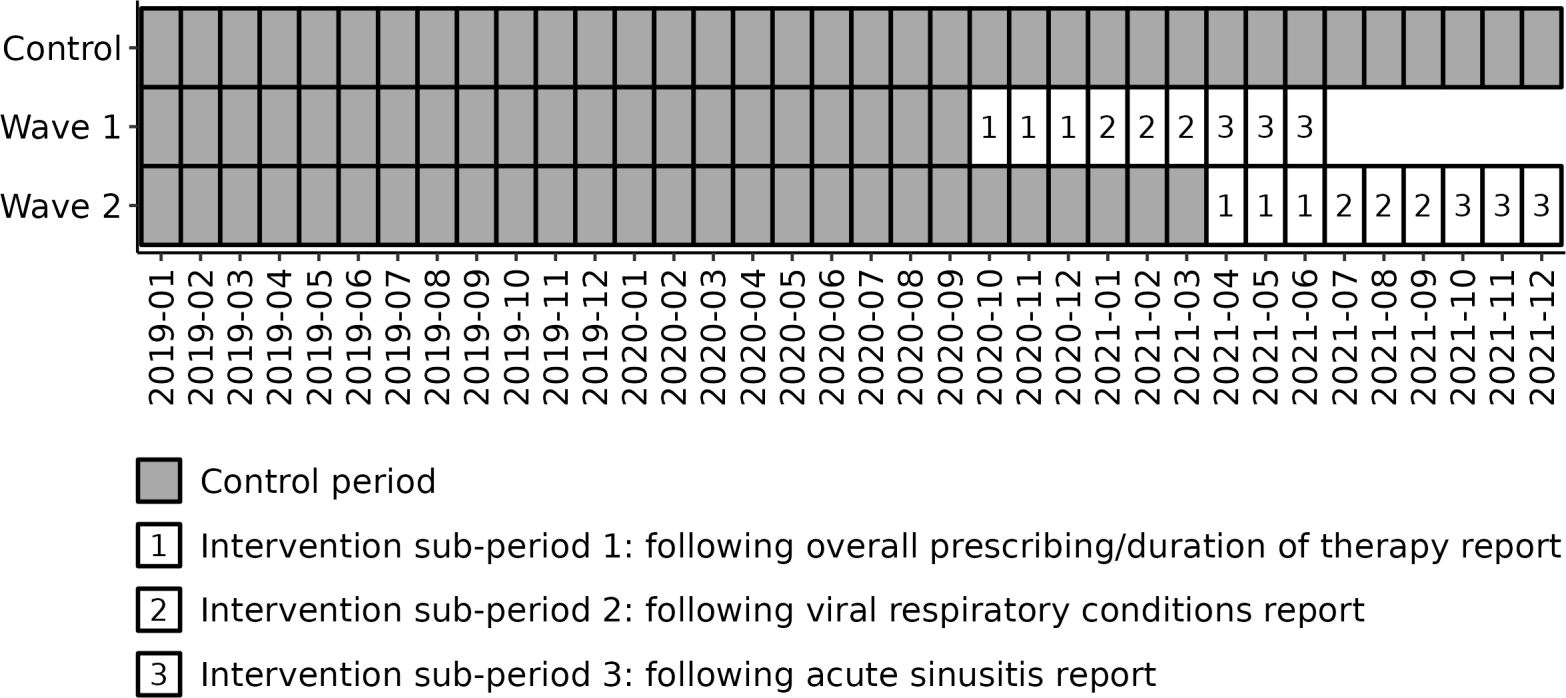
Study timeline showing intervention periods and sub-periods for the two study waves. Intervention wave 1 included only participants from Canada; intervention wave 2 included participants from both Canada and Israel.

The intervention consisted of three personalized reports delivered by email at the beginning of the first, fourth, and seventh months of the intervention period (Fig. 1). Emails were sent to participants individually by collaborators at each study site. Each topic-specific report contained locally relevant guidelines and one or two figures benchmarking the intervention participant’s prescribing against the average and 25th percentile of other intervention participants at the same site (see summary of report contents in Table S1). To generate benchmarking figures, we used prescribing data from 2019.

The first report covered overall prescribing and duration of therapy and included two benchmarking figures: (i) the overall antibiotic prescribing rate per 100 visits and (ii) the percentage of antibiotic prescriptions with a duration greater than 7 days (Canada only). Guidelines described how most outpatient bacterial infections do not require more than 7 days of therapy. The second report targeted viral respiratory conditions and included a benchmarking figure on the antibiotic prescribing rate per 100 visits for viral respiratory conditions. The third report targeted acute sinusitis and included a benchmarking figure on the antibiotic prescribing rate per 100 visits for acute sinusitis. An example report is included as Supplementary File 2.

### Study period

The study period spanned from January 2019 to December 2021. Three reports were sent to intervention participants over the course of the intervention period: one at the beginning of the first month, another at the beginning of the fourth month, and the last at the beginning of the seventh month, with reports spaced three months apart (Fig. 1). We defined each 3-month period following the receipt of a report as an intervention sub-period, for a 9-month total intervention period (Fig. 1). Two waves of intervention participants were recruited: wave 1 (reports sent between October 2020 and April 2021; total intervention period: October 2020–June 2021) included only participants from Canada, whereas wave 2 (reports sent between April 2021 and October 2021; total intervention period: April 2021–December 2021) included participants from both sites (Fig. 1).

### Outcomes

We evaluated four outcomes related to the content of the feedback reports:

1. Overall antibiotic prescribing rate (percentage of total visits with an antibiotic prescription).
2. Mean duration of therapy per antibiotic prescription.
3. Antibiotic prescribing rate for viral respiratory conditions (percentage of visits for viral respiratory conditions with an antibiotic prescription).
4. Antibiotic prescribing rate for acute sinusitis (percentage of visits for acute sinusitis with an antibiotic prescription).

All outcomes were available as aggregated monthly data. Due to data set differences between our two study sites, we defined the duration of therapy in Canada as days of therapy and in Israel as Defined Daily Doses (DDDs). Table S2 gives the ICD-9 codes used to define viral respiratory conditions (and associated indications) and acute sinusitis. Note that COVID-19 was not included in the definition for viral respiratory conditions.

### Data acquisition and cleaning

Details regarding data acquisition and cleaning are given in the Supplementary Methods.

### Statistical analysis

We performed multilevel regression modeling of our four outcomes with terms for site, study month, the interaction between study site and month, and temporal autocorrelation. The effect of the intervention during the 9-month intervention period was estimated as *β*_total_. For overall and indication-specific prescribing, we used logistic models, and for the mean duration of therapy, we used a zero-truncated Poisson model. We fit all models in R version 4.1.3 (30) using maximum likelihood estimation via glmmTMB version 1.1.5 (31). Further details, including the assessment of pre-intervention trends in intervention participants and controls, are described in the Supplementary Methods.

### Sensitivity analyses

We fit an alternative set of models to estimate separate intervention effects for each of the three intervention sub-periods (*β_1_*, *β_2_*, and *β_3_*) (Fig. 1). For Canada only (due to data availability), we considered an alternative metric for the duration of therapy outcome: the percentage of antibiotic prescriptions with a duration greater than 7 days (further described in the Supplementary Methods). For the four main outcomes, we also fit site-stratified models using data from only a single site at a time.

## RESULTS

Complete follow-up data were available for 11 intervention participants and 361 controls in Canada and 21 intervention participants and 364 controls in Israel. Some physicians in Canada received the intervention, but their data could not be included in the analysis (Supplementary Methods). In Canada, intervention participants had higher baseline (2019) antibiotic prescribing rates than controls; they were also younger, more likely to be male, and had more monthly visits (Table 1). In Israel, intervention participants had slightly lower baseline prescribing rates than controls; they were also more likely to be male and had more monthly visits (Table 1). Intervention and control participants showed similar temporal trends in prescribing prior to the intervention period (Fig. S1).

**TABLE 1:** Baseline characteristics of intervention participants (*n* = 32) and controls (*n* = 725) based on 2019 overall antibiotic prescribing data

Group	*n*	Median and Q_1_, Q_3_ of overall prescribing (% of visits)	Mean monthly visits	% male	Mean age
Canada					
Intervention	11	8.4 (6.3, 11.0)	343	54.5	41.0
Control	361	5.2 (3.8, 6.9)	276	38.5	46.5^*a*^
Israel					
Intervention	21	6.5 (4.9, 7.7)	783	61.9	53.9
Control	364	7.4 (5.9, 9.5)	649	51.1	53.2

^*a*^ Age was missing for 21 Canadian controls.

**Fig 2:**
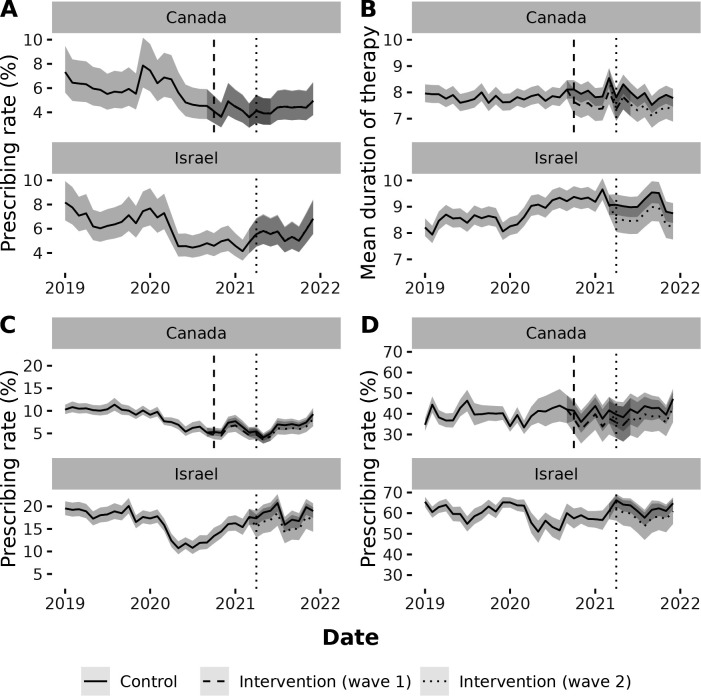
Fitted values for the control period, wave 1 intervention period, and wave 2 intervention period for (A) overall prescribing, (**B**) mean duration of therapy, (**C**) prescribing for viral respiratory conditions, and (**D**) prescribing for acute sinusitis among primary care physicians in Canada and Israel, 2019–2021. Vertical bars identify the beginning of the 9-month intervention periods (wave 1 in Canada and wave 2 in Canada and Israel).

The onset of the COVID-19 pandemic led to large drops in overall antibiotic prescribing per visit in both Canada and Israel, with the prescribing rate from March 2020 to February 2021 dropping by 21.0% and 26.9%, respectively, compared to the same period the year prior (Fig. 2). By the end of 2021, overall prescribing rates were trending toward pre-pandemic levels in Israel but remained depressed in Canada. The prescribing rate for viral respiratory conditions per visit also declined in both sites in 2020. The mean duration of therapy in Israel rose after the onset of the pandemic. Average monthly patient visits per physician dipped in 2020 in Canada but not in Israel (Fig. S2). Visits for viral respiratory conditions and acute sinusitis declined in both sites in 2020 and began to increase in 2021.

Across the 9-month intervention period, overall antibiotic prescribing did not decline among intervention participants (OR = 1.01; 95% CI: 0.94, 1.07) (Fig. 2), but the mean duration of therapy did decline (IRR = 0.94; 95% CI: 0.90, 0.99) (Fig. 2). In the site-stratified sensitivity analysis, this effect appeared to differ between Canada (IRR = 0.89; 95% CI: 0.82, 0.98) (Table S3) and Israel (IRR = 0.97; 95% CI: 0.92, 1.02) (Table S4). There was a trend toward reduced antibiotic prescribing for viral respiratory conditions (OR = 0.87; 95% CI: 0.73, 1.03) and acute sinusitis (OR = 0.85; 95% CI: 0.67, 1.07) (Fig. 2), but neither decline was statistically significant. Effect estimates varied in the sensitivity analysis using three intervention sub-periods (Table S5).

In a sensitivity analysis using Canadian data only, we observed a non-significant reduction in the percentage of antibiotic prescriptions with a duration of 7 days or more (OR = 0.83; 95% CI: 0.68, 1.01) (Table S3), although the direction of the point estimate was consistent with the observed decline in the mean duration of therapy.

## DISCUSSION

We conducted a quasi-experimental study to assess the impact of an automated feedback intervention on antibiotic prescribing. We found that the intervention was associated with a decrease in the mean duration of therapy per prescription. While there was no change in overall prescribing, we did observe non-significant trends toward reduced prescribing for viral respiratory conditions and acute sinusitis. Our data support the use of an open, low-resource, automated feedback tool to improve antibiotic prescribing among primary care physicians.

The reduction in the mean duration of therapy we observed during the intervention period was equivalent to approximately half a day per prescription, which translated to a median reduction of 14 days of therapy (Q_1_, Q_3_: 7, 22) per month during the intervention period compared to what was expected in the absence of the intervention. Since most common infections do not require more than 5–7 days of therapy (32), and the mean duration of therapy per prescription was still greater than 7 days even during the intervention period, it is very likely that this reduction represented a positive improvement for stewardship without compromising patient care. The results of our trial are consistent with the results of other interventions targeting duration of therapy for specific conditions such as community-acquired pneumonia and urinary tract infections (33, 34), or as part of a multifaceted stewardship intervention to reduce overall antibiotic prescribing (35).

A recent meta-analysis of 10 antimicrobial stewardship programs in outpatient/primary care practice settings found a 6% (95% CI: −13%–1%) average reduction in the fraction of patients receiving an antibiotic (36). These interventions included a variety of countries and metrics (such as overall prescribing or prescribing for respiratory infections), including contexts where pre-intervention prescribing rates were significantly higher than those that existed in our study population. Although our intervention was unsuccessful at reducing overall prescribing, the observed reductions in indication-specific prescribing rates, while not statistically significant, imply absolute reductions in prescribing among patients with viral respiratory conditions or acute sinusitis between 1% and 4%. Randomized trials by Hallsworth et al. (14) and Schwartz et al. (15) provide evidence that a simple intervention (a peer comparison letter sent to high-prescribing physicians) can deliver a small but significant reduction in antibiotic prescribing, demonstrating the meaningful role low-resource interventions can play in advancing antimicrobial stewardship.

It is important to recognize that our study took place during a period of unprecedented disruption to primary care due to the COVID-19 pandemic, which began in most parts of the world in March 2020. In the months that followed, many countries transitioned to telemedicine, diverted resources to caring for COVID-19 patients, and experienced disruptions to civil society and workplaces. Contrary to initial fears, this confluence of factors led to a very large reduction in antimicrobial prescribing in primary care in many countries (19, 37–40). These observations are consistent with the large reductions in prescribing following the onset of the COVID-19 pandemic seen in our own study cohort.

These unusual circumstances may have affected our antimicrobial stewardship intervention in several ways. This dramatic reduction in antibiotic prescribing rates in the months before our intervention period may have made physicians less amenable to further reductions in prescribing. Additionally, while a key part of our messaging was focused on prescribing for seasonal viral conditions, the first 2 years of the pandemic were marked by the suppression of many seasonal viruses, most notably the near-total disappearance of the 2020–2021 influenza season (41, 42).

We must also acknowledge several other limitations that may have impacted our findings. First, recruitment was more difficult than anticipated due to the onset of the COVID-19 pandemic, and a greater number of intervention participants may have added precision to our effect estimates. Second, we estimated a common intervention effect across study sites, although the type of participants in the intervention groups may have differed (in Canada, intervention participants had higher than average baseline prescribing, whereas in Israel, they had slightly lower than average prescribing). We were also unable to deliver the peer benchmarking figure for the duration of therapy in the first intervention report to Israeli participants (Supplementary Methods). These factors may have resulted in heterogeneity in the intervention effects. Finally, our use of two study waves meant that some study reports, particularly those related to prescribing for viral respiratory conditions, may not have been delivered at the optimal time (cold and influenza season). However, the importance of this was unclear due to the disruption of regular seasonal trends of respiratory viruses during the COVID-19 pandemic.

While our intervention did not demonstrate a reduction in overall prescribing, we did observe a decrease in the mean duration of therapy per prescription, an important component for a successful antimicrobial stewardship program (43). As the drought of new antibiotic agents persists, the need for innovative solutions for antimicrobial stewardship, particularly within primary care, is more critical than ever. The ability to track, evaluate, and report on antimicrobial prescribing is a core element of outpatient stewardship (17). Despite the proven success of bespoke interventions for reducing antimicrobial prescribing, the technical capacity to carry out the basic activities of stewardship in a sustainable way remains a significant barrier to long-term progress in implementing best practices in the community setting (23, 24). OPEN Stewardship is part of a new generation of accessible, low-resource tools for advancing stewardship across heterogeneous, resource-constrained outpatient health care settings (another example is OASIS (44), which uses the common statistical software SAS). Going forward, it is critical that we continue to develop and deploy automated, scalable antimicrobial stewardship interventions targeted at reducing outpatient prescribing, in order to safeguard the efficacy of antimicrobial therapy into the future.

## Data Availability

Primary care data relating to physicians and their patients cannot be shared in compliance with our agreements with our data partners (UTOPIAN and Clalit Health Services). However, other parties can apply to UTOPIAN and Clalit Health Services for research data access.

## SUPPLEMENTAL MATERIAL

Supplemental material spectrum.00017-24-s0001.pdf Supplemental methods, tables, and figures.

Supplemental File 2 spectrum.00017-24-s0002.pdf An example of an OPEN Stewardship report.

ASM does not own the copyrights to Supplemental Material that may be linked to, or accessed through, an article. The authors have granted ASM a non-exclusive, world-wide license to publish the Supplemental Material files. Please contact the corresponding author directly for reuse.
