# Supplementary material for: Evaluation of an automated feedback intervention to improve antibiotic prescribing among primary care physicians (OPEN Stewardship): a multinational controlled interrupted time-series study
Source: Microbiol Spectr. 2024 Feb 27;12(4):e00017-24. doi: 10.1128/spectrum.00017-24 (PMC10986525; doi:10.1128/spectrum.00017-24)

Supplementary Material: Evaluation of an automated feedback intervention to improve antibiotic prescribing among primary care physicians (OPEN Stewardship): a multinational controlled interrupted time-series study

Jean-Paul R. Soucy, Marcelo Low, Kamal R. Acharya, Moriah Ellen, Anette Hulth, Sonja Löfmark, Gary E. Garber, William Watson, Jacob Moran-Gilad, Nadav Davidovich, Tamar Amar, Janine McCready, Matthew Orava, John S. Brownstein, Kevin A. Brown, David N. Fisman, Derek R. MacFadden

Correspondence to: Dr. Derek R. MacFadden (dmacfadden@toh.ca)

SUPPLEMENTARY METHODS

Study enrollment and follow-up

In Canada, primary care physicians in the Greater Toronto Area were offered enrollment via email or presentations to groups of providers and were considered eligible if their prescribing data were available in the UTOPIAN database. In Israel, half of primary care physicians within Clalit Health Services (192 physicians) were offered enrollment via a letter from the chief physician, with follow-up by an interviewer.

In total, we enrolled 43 physicians in total to the intervention, 22 from Canada and 21 from Israel. In Canada, all 22 intervention participants received the intervention, but a data access issue arising during the analysis phase rendered follow-up prescribing data unavailable for 11/22 participants and an unknown number of controls. In total, complete follow-up data were available for 11 intervention participants and 361 controls in Canada and 21 intervention participants and 364 controls in Israel. Of the 11 Canadian intervention participants with available data, 4 belonged to wave 1 (beginning October 2020) and 7 belonged to wave 2 (beginning April 2021); all 21 intervention participants from Israel belonged to wave 2.

Data acquisition

Monthly aggregated antibiotic prescribing data by provider were acquired from the University of Toronto Practice-Based Research Network (UTOPIAN) for Canada and Clalit Health Services (CHS) for Israel. Israeli data covered visits by adults (aged 18+) insured by CHS. For each provider-month, the following data were available on antibiotic prescribing:

- The overall number of prescriptions
- The number of prescriptions for the study-defined indication categories
- The overall number of visits
- The number of visits for the study-defined indication categories
- The total duration of therapy for all prescriptions
- The overall number of prescriptions with duration greater than 7 days (Canada only)

The ICD-9 codes corresponding to the study-defined indication categories, viral respiratory conditions (and associated indications) and acute sinusitis, are detailed in Supplementary Table

2. Prescriptions were linked to visits based on local linkage algorithms, and many prescriptions could not be linked to a specific indication (but were included in the overall prescription numbers). Due to differences in the underlying datasets, duration of therapy was defined as days of therapy in the Canadian dataset and Defined Daily Doses (DDDs) in the Israeli dataset. Missing data for duration of therapy was handled locally: filtered out in Canada and imputed based on default values in Israel. Finally, we obtained basic demographic characteristics (age and sex) for participating and non-participating providers.

Survey data were also collected from intervention participants. One month after receiving each of the three reports, intervention participants received a survey to capture their thoughts on the intervention. The results of these surveys will be reported in a forthcoming manuscript.

Data cleaning

The combined datasets (which included data from Canada and Israel for both intervention and control participants) were cleaned to remove implausible/likely erroneous values. For the overall prescribing dataset, we removed 129 provider-months where the average duration of therapy per prescription exceeded six weeks, as these represent either extremely atypical community prescribing or data errors. For all datasets, we removed 18 provider-months where the number of prescriptions exceeded the number of visits (all observations were in the overall prescribing dataset). Most of these observations implied ≥2.5 prescriptions per visit, which likely indicated errors in how visits were calculated in those provider-months.

Statistical analysis

For the overall and indication-specific prescribing outcomes, we fit multilevel logistic regression models including terms for the study site (Canada versus Israel), study month (e.g., November 2020, July 2021, etc.), and the interaction between study site and study month. This model specification accounts for the temporal trends in prescribing that may differ across study sites. Since the vast majority of primary care visits will result in one or zero prescriptions, we performed logistic regression using the number of prescriptions for a particular outcome as the number of successes and the number of visits (overall or indication-specific, depending on the outcome) as the number of trials. Random intercepts for each provider, nested within the study group (Canada intervention wave 1, Canada intervention wave 2, Canada control, Israel intervention wave 2, Israel control), nested within the study site, were included. Finally, we added an AR(1) term to account for temporal autocorrelation among the residuals for each provider.

For the mean duration of therapy outcome, we fit a zero-truncated Poisson model using the same model structure but with total duration of therapy as the outcome and the log of total prescriptions as an offset. A zero-truncated negative binomial model was considered but was rejected by comparing model AIC values.

We fit a pair of models for each of the four outcomes. For the total intervention period model, we created a binary variable taking a value of 1 for months within the nine-month intervention period (for intervention participants) and a value of 0 otherwise (Figure 1). This coefficient (β_{total}) estimates the deviation from the expected outcome that is attributable to the intervention.

Participants in wave 1 of the intervention were censored following the end of their intervention period (to avoid contaminating the baseline expectation); the end of the intervention period in wave 2 coincided with the end of the study. To examine each of the three three-month intervention sub-periods, we fit an alternative set of models with three intervention effects, one for each sub-period (β_1, β_2, β_3). We created three binary variables corresponding to the three three-month intervention periods: these take a value of 1 for months within the respective intervention sub-period (for intervention participants) and a value of 0 otherwise (Figure 1).

Model residuals were assessed using the R (1) package *DHARMa* version 0.4.6 (2).

Assessment of pre-intervention trends

We assessed the similarity of prescribing trends in intervention and control participants prior to the intervention period by fitting an additional set of models. Within the two study sites, models were fit for each of the four study outcomes using data beginning January 2019 and ending the month prior to the first intervention wave in the site (September 2020 in Canada and March 2021 in Israel). The multilevel regression models included terms for enrollment status (intervention versus control), study month, and the interaction between enrollment status and study month. Random intercepts for each provider, nested within enrollment status, were included, as was an AR(1) term to account for temporal autocorrelation among the residuals for each provider.

To compare the pre-intervention trends for each region-outcome pair, we plotted the predicted values of intervention and control participants and visually assessed their similarity.

Sensitivity analyses

For Canada only (due to data availability), we considered an alternative metric for the duration of therapy outcome: the percentage of antibiotic prescriptions with a duration greater than 7 days. We fit a logistic regression model using the number of prescriptions with duration greater than 7 days as the number of successes and the total number of prescriptions as the number of trials.

Summary of changes from protocol

Changes to intervention

Since data on days of therapy was only available for Canada (Israel provided DDDs as its measure of duration of therapy), the first intervention report was different between the two study sites. As detailed in Supplementary Table 1, the first intervention report was intended to include peer benchmarking figures for both the overall antibiotic prescribing rate and the percentage of prescriptions with duration greater than 7 days. However, the latter figure could not be generated for Israeli participants and was thus included in the first intervention report only for Canadian participants.

Changes to study timeline

In the original protocol, the study timeline figure displayed a 12-month control period prior to the intervention as an illustration; however, to accurately capture each provider's baseline prescribing rate, we used prescribing data beginning from January 2019 in the analysis (Figure 1). For each study participant, their intervention reports were generated using their prescribing data from January 2019–December 2019, as described in the original protocol.

The evaluation periods for each outcome were initially described as being the six months following the receipt of the report targeting that outcome. However, this had two issues: 1) the three reports are all components of a single intervention, rather than three independent interventions and 2) for reports two and three, the "control" period preceding the evaluation period was contaminated by previous reports (e.g., for the second report on prescribing for viral respiratory conditions, participants had already received the first report targeting their overall prescribing; however, the three months between the receipt of the first and second reports was considered part of the control period for evaluating the viral respiratory condition prescribing outcome). Instead, in this analysis, we define three intervention sub-periods of three months each following the receipt of each intervention report and define the total nine-month period as the intervention period (Figure 1). With the view that each report forms an incremental part of the whole stewardship intervention designed to improve appropriate antibiotic prescribing, we fit models to estimate the intervention effects for all outcomes 1) during the total nine-month intervention period and 2) during the three three-month intervention sub-periods, as detailed in the *Statistical analysis* section of the main manuscript.

Changes to statistical analysis

Prior to the COVID-19 pandemic, we anticipated that the temporal trends in antibiotic prescribing would follow a predictable seasonal cycle with a gentle longer-term trend. However, it later became clear that the COVID-19 pandemic and evolving response to it was driving the dramatic changes in prescribing rates that we observed during the study period. Thus, the statistical models were simplified and re-focused on estimating the temporal trends at each site via the inclusion of a term for study month (e.g., November 2020, July 2021, etc.) with an interaction for the study site, as described in the *Statistical analysis* section of the main manuscript.

The protocol originally proposed negative binomial models for prescribing rate outcomes (overall and indication-specific), using the log of the number of visits as the offset in the model. However, since the vast majority of visits in primary care result in either zero or one antibiotic prescriptions, the logistic regression model better captures the underlying distribution of the outcome. While it is theoretically possible for the number of antibiotic prescriptions to exceed the number of visits in a month (an observation incompatible with the logistic regression model), most observations of this nature in our dataset clearly result from erroneous data (as described in the *Data cleaning* section above).

Since the guidelines in the first report specifically targeted prescribing with duration greater than

7 days, the protocol originally proposed the outcome of the percentage of prescriptions with duration greater than 7 days as the primary model (logistic regression) and the outcome of the mean duration of therapy as an alternative model (zero-truncated Poisson regression). However, since the data to perform the former analysis was only available for Canada, we used the mean duration of therapy model as the primary analysis for this prescribing metric. The zero-truncated Poisson model also preserves more information by avoiding dichotomization of the outcome, improving power.

References

1. R Core Team. R: A Language and Environment for Statistical Computing [Internet]. Vienna, Austria: R Foundation for Statistical Computing; 2021. Available from: https://www.R-project.org/

2. Hartig F. DHARMa: Residual Diagnostics for Hierarchical (Multi-Level / Mixed) Regression Models [Internet]. 2022. Available from: https://CRAN.R-project.org/package=DHARMa

SUPPLEMENTARY TABLES

Supplementary Table 1. Composition of intervention feedback reports at each time point.

Report	Peer benchmarking figures[1]	Local guidelines
Overall prescribing/duration of therapy (t_0)	- Overall antibiotic prescribing rate per 100 visits<br>- Percentage of antibiotic prescriptions with duration greater than 7 days[2]	Guidelines indicating that antibiotic prescribing longer than 7 days is not necessary for many conditions
Viral respiratory conditions (t_3)	- Antibiotic prescribing rate for viral respiratory conditions per 100 visits for viral respiratory conditions	Prescribing guidelines for viral respiratory conditions
Acute sinusitis (t_6)	- Antibiotic prescribing rate for acute sinusitis per 100 visits for acute sinusitis	Prescribing guidelines for acute sinusitis

[1] In each peer benchmarking figure, the intervention participant's own rate/percentage is compared to the average and 25th percentile rate/percentage of all intervention participants at the same study site using prescribing data from 2019.

[2] This figure was only presented in Canadian reports because data on days of therapy were not available for Israel.

Supplementary Table 2. ICD-9 codes for indications identified as viral respiratory conditions and acute sinusitis in the OPEN Stewardship platform.

OPEN Stewardship	Indication category	ICD-9 codes
Viral respiratory conditions	Asthma/allergy	477 Allergic rhinitis, 493 Asthma, 995.3 Allergy, unspecified
	Bronchitis/bronchiolitis	466 Acute bronchitis and bronchiolitis, 490 Bronchitis, not specified as acute or chronic, 492 Emphysema, 496 Chronic airway obstruction, not elsewhere classified
	Influenza	487 Influenza, 488 Influenza due to identified avian influenza virus
	Viral pneumonia	480 Viral pneumonia
	Viral upper respiratory tract infection	460 Acute nasopharyngitis [common cold], 464 Acute laryngitis and tracheitis, 465 Acute upper respiratory infections of multiple or unspecified sites, 786.2 Cough
Acute sinusitis	Acute sinusitis	461 Acute sinusitis

Supplementary Table 3. Intervention effect estimates with 95% confidence intervals for the total intervention period and each of the three three-month intervention sub-periods for models fit to the Canadian data alone.

**Outcome**	β_{total}	β_1	β_2	β_3
Overall prescribing[1]	1.03 (0.91, 1.17)	1.04 (0.88, 1.23)	0.96 (0.80, 1.15)	1.08 (0.90, 1.29)
Duration of therapy[2]	0.89 (0.82, 0.98)	0.97 (0.84, 1.11)	0.80 (0.69, 0.93)	0.91 (0.79, 1.05)
Viral respiratory conditions[1]	0.90 (0.64, 1.27)	0.66 (0.39, 1.11)	0.78 (0.40, 1.51)	1.34 (0.83, 2.15)
Acute sinusitis[1]	0.95 (0.54, 1.67)	1.25 (0.40, 3.88)	0.70 (0.32, 1.51)	1.36 (0.49, 3.76)
Prescriptions with duration greater than 7 days[1]	0.83 (0.68, 1.01)	0.88 (0.66, 1.17)	0.78 (0.58, 1.06)	0.81 (0.60, 1.10)

[1] Odds ratio from a logistic regression model
[2] Incidence rate ratio from a zero-truncated Poisson model

Supplementary Table 4. Intervention effect estimates with 95% confidence intervals for the total intervention period and each of the three three-month intervention sub-periods for models fit to the Israeli data alone.

**Outcome**	β_{total}	β_1	β_2	β_3
Overall prescribing[1]	0.99 (0.92, 1.06)	1.01 (0.93, 1.10)	1.00 (0.91, 1.09)	0.94 (0.85, 1.03)
Duration of therapy[2]	0.97 (0.92, 1.02)	0.96 (0.89, 1.04)	0.96 (0.89, 1.03)	0.99 (0.92, 1.06)
Viral respiratory conditions[1]	0.87 (0.71, 1.07)	0.81 (0.64, 1.04)	0.79 (0.59, 1.07)	1.00 (0.77, 1.31)
Acute sinusitis[1]	0.85 (0.67, 1.09)	0.65 (0.46, 0.93)	1.03 (0.68, 1.57)	1.00 (0.69, 1.45)

[1] Odds ratio from a logistic regression model
[2] Incidence rate ratio from a zero-truncated Poisson model

Supplementary Table 5. Intervention effect estimates with 95% confidence intervals for the total intervention period and each of the three three-month intervention sub-periods.

**Outcome**	β_{total}	β_1	β_2	β_3
Overall prescribing[1]	1.01 (0.94, 1.07)	1.02 (0.95, 1.11)	0.99 (0.91, 1.09)	0.98 (0.90, 1.08)
Duration of therapy[2]	0.94 (0.90, 0.99)	0.96 (0.90, 1.04)	0.90 (0.84, 0.97)	0.96 (0.89, 1.03)
Viral respiratory conditions[1]	0.87 (0.73, 1.03)	0.79 (0.63, 0.98)	0.78 (0.60, 1.01)	1.04 (0.83, 1.30)
Acute sinusitis[1]	0.85 (0.67, 1.07)	0.68 (0.48, 0.95)	0.92 (0.64, 1.32)	1.02 (0.72, 1.45)

[1] Odds ratio from a logistic regression model
[2] Incidence rate ratio from a zero-truncated Poisson regression model

SUPPLEMENTARY FIGURES

Supplementary Figure 1. Fitted values for intervention and control participants for A) overall prescribing, B) mean duration of therapy, C) prescribing for viral respiratory conditions, D) prescribing for acute sinusitis among primary care physicians in Canada and Israel during the pre-intervention period (January 2019 to September 2020 for Canada and January 2019 to March 2021 for Israel).

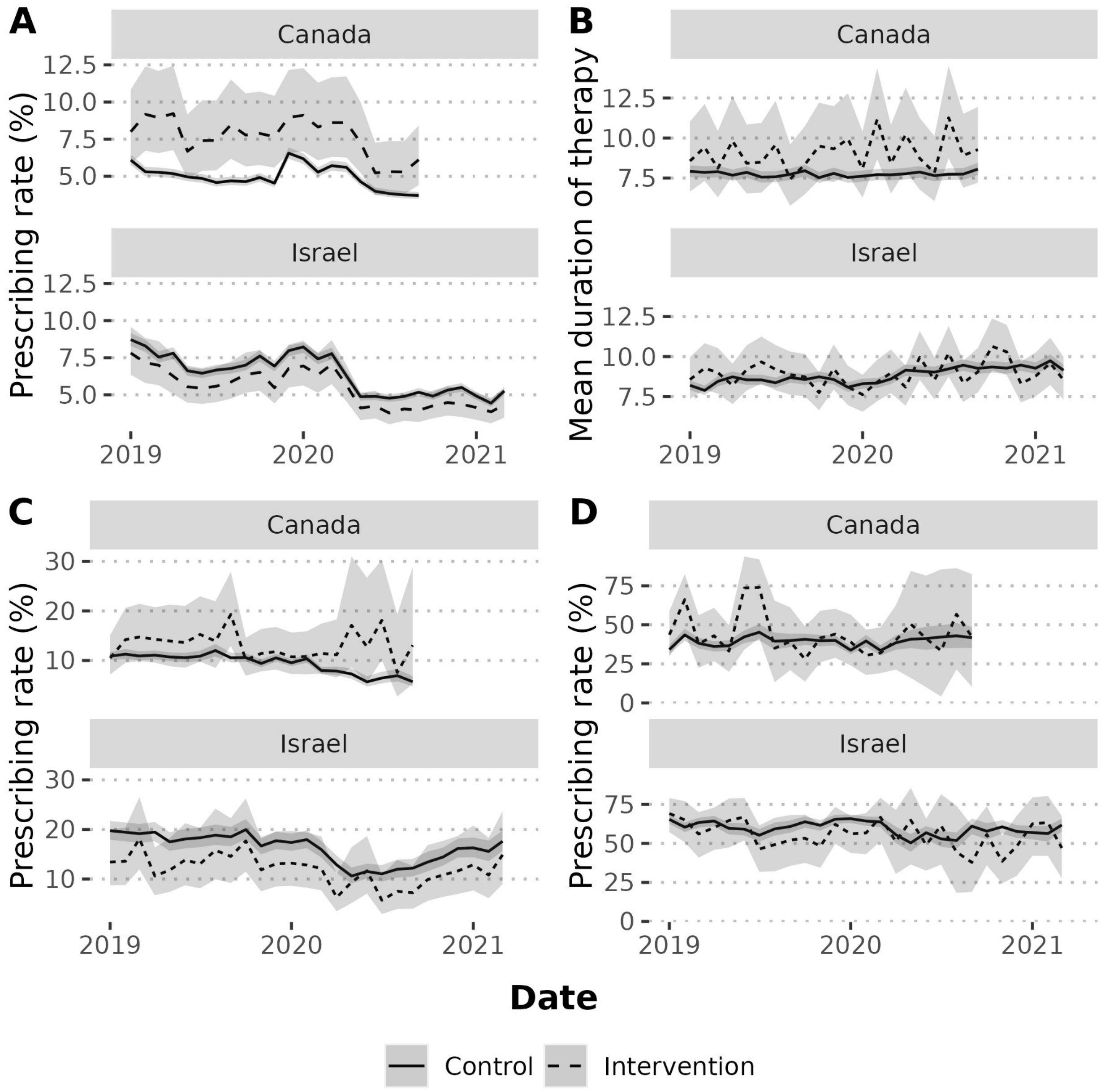

Supplementary Figure 2. Monthly number of visits per provider for A) overall visits, B) visits for viral respiratory conditions, C) visits for acute sinusitis among primary care physicians in Canada and Israel, 2019–2021. Trend shown as loess curve with 95% confidence interval.

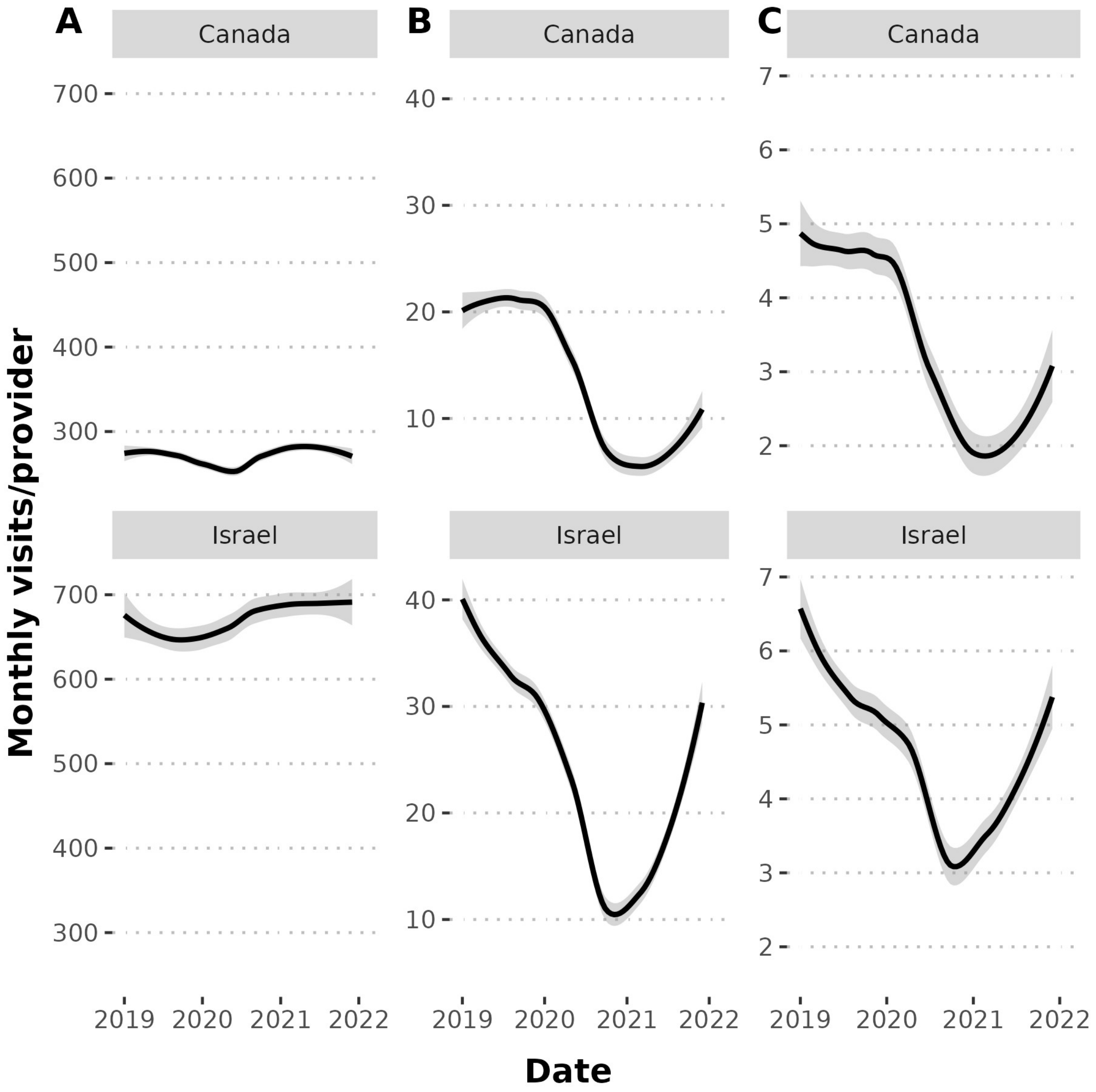

Supplement: Supplemental material — Supplemental methods, tables, and figures. [file spectrum.00017-24-s0001.pdf]
