# Supplementary material for: Evaluation of an automated feedback intervention to improve antibiotic prescribing among primary care physicians (OPEN Stewardship): a multinational controlled interrupted time-series study
Source: Microbiol Spectr. 2024 Feb 27;12(4):e00017-24. doi: 10.1128/spectrum.00017-24 (PMC10986525; doi:10.1128/spectrum.00017-24)
Supplement: Supplemental File 2 — An example of an OPEN Stewardship report. [file spectrum.00017-24-s0002.pdf]

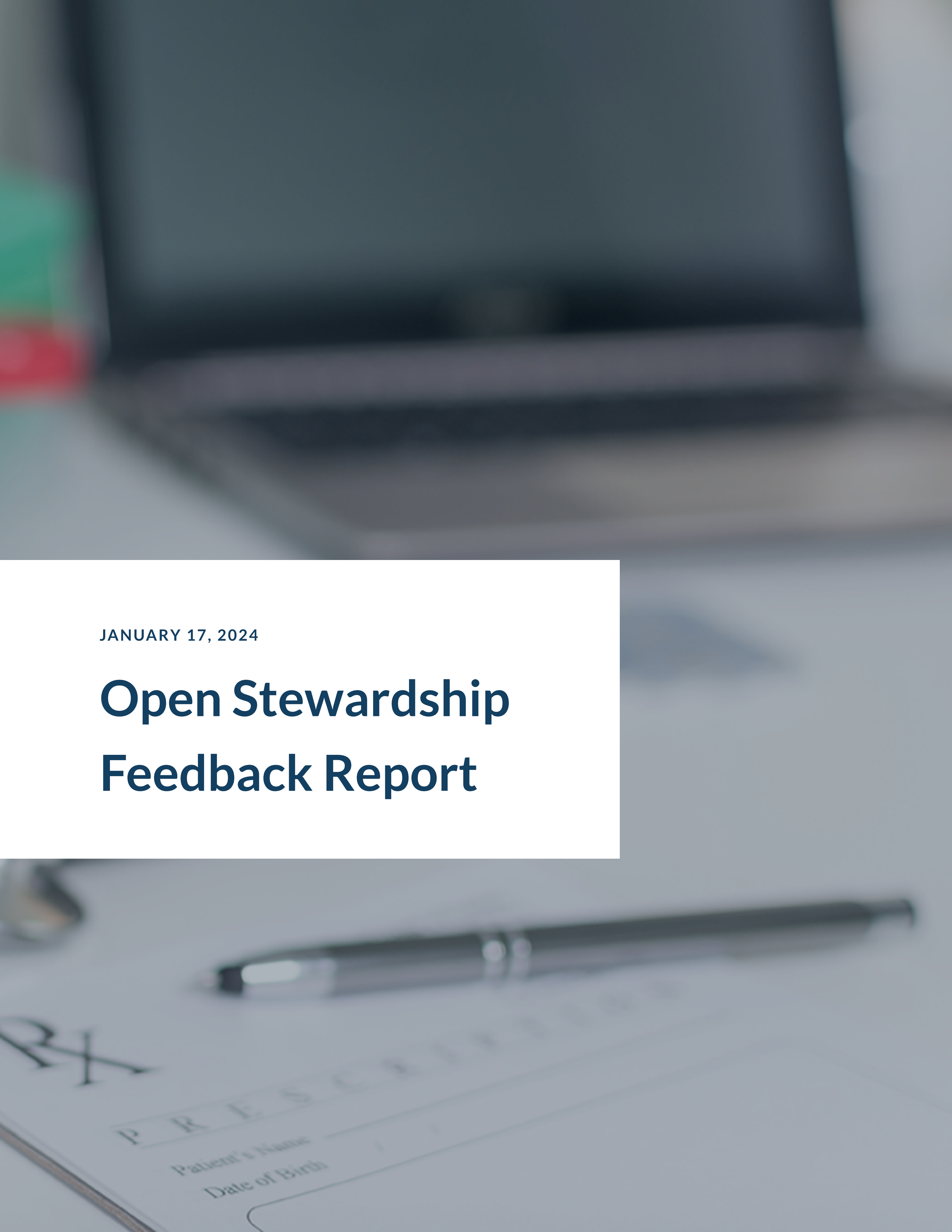

JANUARY 17, 2024

Open Stewardship Feedback Report

Number of Prescriptions of Any Antibiotic (Per 100 Visits) for 'Acute sinusitis'

You prescribed 17% below average

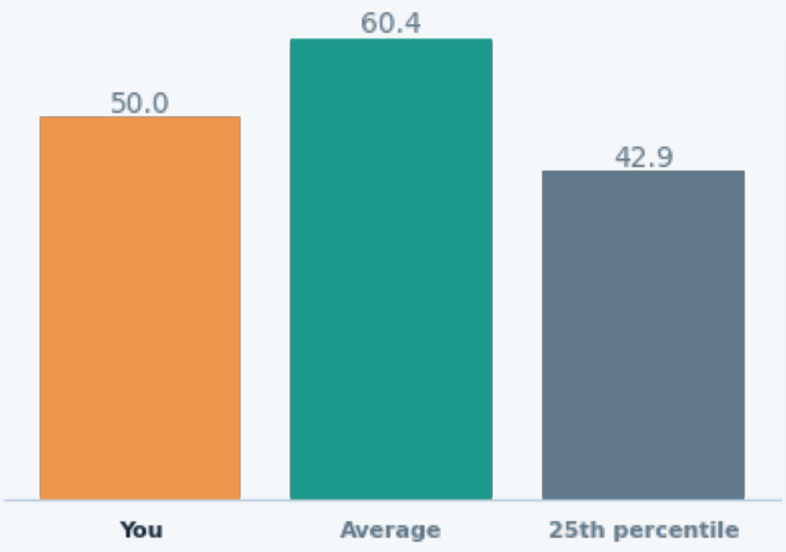

You had 40 patient visits from Jan 01, 2019 to Dec 31, 2019 .

This figure shows how frequently you prescribed antibiotics for acute sinusitis in 2019. Your prescribing rate (prescriptions per 100 visits) is compared to the average (mean) of your participating colleagues in Southern Ontario as well as showing your position relative to the 25th percentile. Generally, it is better to be below the average rate of prescribing and within the lowest 25th percentile.

Please note that discrepancies in the number of visits recorded for acute sinusitis likely result from differences in coding practices.

These data are not adjusted for physician's practice characteristics, nor do they differentiate between delayed or non-filled prescriptions. These results are generated from claims data, and misclassification of the diagnosis may be present. As a result, our prescribing targets are referenced to peer benchmarks. Learn more about the data and its interpretation.

Acute sinusitis (Adult)

COMMON PATHOGENS

Viruses

Streptococcus pneumoniae (non-meningitis)

Haemophilus influenzae

Moraxella sp.

RECOMMENDED TREATMENT

First Line

Treatment Regimen Option

Drug	Dose	Frequency	Route	Duration
**No Antibiotic Treatment Indicated**	. mg	OD	PO	. days

Most acute sinusitis is viral and does not require antibiotics. Antibiotics may be considered if symptoms are not improving after 10 days, are severe (>3 days), or improved then worsened and lasting >3 days. See below for 1st line therapy if indicated.

Treatment Regimen Option

Drug	Dose	Frequency	Route	Duration
**Amoxicillin**	500 mg	TID	PO	5-7 days

Second Line

Treatment Regimen Option

Drug	Dose	Frequency	Route	Duration
**Amoxicillin-Clavulanic acid**	875/125 mg	BID	PO	5-7 days

GENERAL COMMENTS

Consider alternative treatment if worsening after 48–72 hours of empiric antimicrobial therapy, or fail to improve despite 3–5 days of therapy.
